# Prognostic value of the duke activity Status Index Questionnaire in predicting mortality in patients with chronic heart failure: 36-month follow-up study

**DOI:** 10.1186/s12872-024-04218-x

**Published:** 2024-10-01

**Authors:** Aldair Darlan Santos-de-Araújo, Daniela Bassi-Dibai, Izadora Moraes Dourado, Renan Shida Marinho, Renata Gonçalves Mendes, Cássia da Luz Goulart, Polliana Batista dos Santos, Meliza Goi Roscani, Shane A. Phillips, Ross Arena, Audrey Borghi-Silva

**Affiliations:** 1 Cardiopulmonary Physiotherapy Laboratory, Universidade Federal de São Carlos, Federal University of Sao Carlos Rodovia Washington Luiz, São Carlos, 13565-905 SP Brazil; 2 Management in Health Programs and Services, Universidade CEUMA, São Luís, MA Brazil; 3 Inter-units of Bioengineering, University of São Paulo, São Carlos, SP Brazil; 4 Health Sciences and Technologies, Universidade de Brasília, Brasília, DF Brazil; 5 Morgana Potrich Faculty, Mineiros, GO Brazil; 6 Department of Medicine, Universidade Federal de São Carlos (UFSCar), Sao Carlos, SP Brazil; 7 Department of Physical Therapy, College of Applied Health Sciences, University of Illinois, Chicago, IL USA

**Keywords:** Chronic heart failure, Duke Activity Status Index, Questionnaire, Mortality, Prognostic

## Abstract

**Background:**

The Duke Activity Status Index (DASI) questionnaire has been the focus of numerous investigations - its discriminative and prognostic capacity has been continuously explored, supporting its use in the clinical setting, specifically during rehabilitation in patients with chronic heart failure (CHF).However, studies exploring optimal DASI questionnaire threshold scores are limited.

**Objective:**

To investigate optimal DASI questionnaire thresholds values in predicting mortality in a CHF cohort and assess mortality rates based on the DASI questionnaire using a thresholds values obtained.

**Methodology:**

This is a prospective cohort study with a 36-month follow-up in patients with CHF. All patients completed a clinical assessment, followed by DASI questionnaire, pulmonary function, and echocardiography. The Receiver Operating Characteristic (ROC) curve analysis was used to discriminate the DASI questionnaire score in determining the risk of mortality. For survival analysis, the Kaplan-Meier model was used to explore the impact of ≤/>23 points on mortality occurring during the 36-month follow-up.

**Results:**

One hundred and twenty-four patients were included, the majority being elderly men. Kaplan Meier analysis revealed that ≤/> 23 was a strong predictor of CHF mortality over a 36-month follow-up.

**Conclusion:**

A score of ≤/>23 presents good discriminatory capacity to predict mortality risk in 36 months in patients with CHF, especially in those with reduced or mildly reduced ejection fraction. Age, ejection fraction, DASI questionnaire score and use of digoxin are risk factors that influence mortality in this population.

## Introduction

Traditionally considered as a multifaceted syndrome due to its impact on organic systems, quality of life, functional capacity and costs to the healthcare system, chronic heart failure (CHF) continues to be closely associated with premature morbidity and mortality [1, 2]. The increase in the global prevalence of CHF has continually challenged researchers and clinicians to optimize its management and detection in its early stages in order to optimally manage the symptomatologic impact [2]. Its heterogeneity and complexity have significant consequences - a key indicator of the burden of CHFis a decrease in exercise and functional capacity [3], associated with an increased risk of hospitalization [4], hospital readmission within thirty days [5] and mortality [6]. In this context, the assessment of exercise and functional capacity is of primary importance in the CHF population [7]. While cardiopulmonary exercise testing is the gold-standard approach to assessing cardiorespiratory fitness, a paradigm that incorporates exercise capacity, its utilization is not always feasible [8]. As such, other approaches quantify exercise and functional capacity in a valid and reliable manner in the CHF population holds clinical relevance [9, 10].

The Duke Activity Status Index(DASI)questionnaire [11]was developed with the aim of offering an efficient, accurate subjective assessment of exercise and functional capacity in individuals diagnosed with cardiovascular disease. Since its creation, the DASI questionnaire has been the subject of several investigations and its discriminative and prognostic capacity has been continuously explored, supporting its use in the clinical setting [12–16].

Approximately 10 years ago, a study investigated the prognostic value (i.e., 5 year mortality term mortality)of the DASI questionnaire in stable patients with CHF [16]. However, the authors did not attempt to identify an optimal cutoff point for the DASI and the analysis only followed the risk of mortality considering the interquartile ranges, without considering the discriminative capacity that evaluate the diagnostic performance of the test. To addressed these unexplored issues, to the current study attempted to investigate optimal DASI questionnaire thresholds values in predicting mortality in a CHF cohort and assess mortality rates based on the DASI questionnaire using a thresholds values obtained.

## Methodology

### Study design

This is a prospective longitudinal study with a three-year follow-up (36 months) carried out at the Cardiopulmonary Physiotherapy Laboratory (LACAP) of the Federal University of São Carlos - UFSCar, São Carlos - SP (Brazil). The ethics committee of the respective university approved the development of the investigation under protocol number 5.188.654and was conducted in accordance with the principles established in the Declaration of Helsinki. The STrengthening the Reporting of OBservational Studies in Epidemiology (STROBE) guideline [17] was used to guide this investigation. Initially, all participants were duly informed about the research objectives and their informed consent was obtained in advance. The study period was from December 01, 2017, through November30, 2023.The participant recruitment period was from December 1, 2017 to December 31, 2023. To ensure an adequate follow-up time of thirty-six months for analysis, the participant search period was from December 1, 2017 to December 31, 2020.In this study, a convenience sampling approach was used. All participants who were invited and met the inclusion criteria were included in the sample.

### Participants

Eligible participants were recruited from the Cardiology Outpatient Clinics of the Medical Specialties Center (CEME) and the São Carlos University Hospital (HU-UFSCar). The following eligibility criteria were adopted: (1) participants of both sexes, aged > 40 years; (2) diagnosed with CHF; (3) left ventricular ejection fraction < 50% previously confirmed by echocardiography; and (4) clinically stable and without medication changes in last three months. The following served as exclusion criteria in the current investigation: (1) aged over 80 years; (2) diagnosed with HF with preserved ejection fraction; (3) history of recent cardiac events (e.g. myocardial infarction and cardiac surgery) in the last 6 months; (4) decompensation of the disease in the three months prior to the start of the investigation; (5) implantable pacemaker; (6) unstable angina; (7) diagnosis of lung cancer or other types of malignant neoplasms; (8) uncontrolled systemic arterial hypertension; (9) uncontrolled diabetes mellitus; (10) cognitive impairment or deficiencies in understanding the study proposal; and 11) refusal to participate in the study.

### Anthropometric variables

A stadiometer (Welmy R-110, Santa Bárbara do Oeste, São Paulo, Brazil) was used to estimate the height of barefoot participants. Body mass in kilograms (kg), body fat mass (kg), percent body fat (%) and skeletal muscle mass (kg) were determined through bioelectrical impedance analysis, using the InBody 720 device. Patients were instructed to fast for at least 4 h, wear light clothing, remove all metallic objects in contact with the body, urinate before the exam, not drink alcohol 12 h before and not perform strenuous exercise the day before the evaluation. The examination consisted of participants positioned in an upright position, barefoot, with shoulders slightly abducted and elbows flexed at approximately 15° as recommended by the manufacturer (BIOSPACE, 2004). The Body Mass Index (BMI) was calculated by dividing body mass (kg) by height squared in meters (kg/m²). The BMI classification was established as follows: low weight (15–19.9 kg/m²); normal weight (20–24.9 kg/m²); overweight (25–29.9 kg/m²); obesity I (30–34.9 kg/m²); obesity II (35–39.9 kg/m²); and obesity III (≥ 40 kg/m²) [18].

### DASI questionnaire

This is a simple and easy-to-understand questionnaire, validated for the Brazilian population [19], consisting of 12 items that evaluate daily activities such as personal care, walking, household chores, sexual activity and recreational activities, estimating their respective metabolic costs. Briefly, the purpose of the questionnaire is to calculate an estimate of maximum oxygen consumption (V̇O_2_) that reflects functional capacity. Each item has a score proportionally based on the metabolic equivalent (MET) of each activity. The final score varies between 0 and 58.2 points, with the higher the score, the better the functional capacity. The V̇O_2_ estimate based on the participantresponses was calculated using the following multiple linear regression equation[11]: V̇O_2_ = 0.43 × DASI + 9.6.

The questionnaire was administered by the researchers involved in the investigation. The administration took place in a private room, ensuring that the environment was controlled and free from distractions. Prior to the collections, the group of researchers was properly trained on all assessments. The training included guidance on how to ask questions consistently, how to record responses correctly, and how to deal with possible doubts or difficulties from participants. In addition, practice tests were carried out to ensure that all administrators were familiar with the questionnaire and followed the same protocol. To minimize measurement bias, the questions were read exactly as written, without interpretations or paraphrasing, to avoid influencing participants’ responses.

### Minnesota living with heart failure questionnaire

The Minnesota Living with Heart Failure Questionnaire (MLHFQ) consists of 21 questions regarding limitations that are often associated with heart failure over the past month [20]. The answers to each question vary on a six-point Likert scale (0–5) representing different degrees of impact of CHF on health-related quality of life, ranging from 0 (no impact) to 5 (very high impact). The questions involve aspects that evaluate the physical dimension (1–7, 12 and 13), which are highly related to dyspnea, fatigue; emotional dimension (17–21); and other questions (numbers 8, 9, 10, 11, 14, 15 and 16) which, together with the previous dimensions, form the total score. This questionnaire has already been previously validated for the Brazilian population [21].

### New York heart association – NYHA

The NYHA classification is a simple, low-cost and well-known functional stratification instrument for CHF, with prognostic value [22, 23]. It is a subjective measure of the physical limitation of a patient with CHF based on the assessment of symptoms and composed of four classes ranging from I to IV: I - absence of symptoms (dyspnea) during daily activities; II - mild symptoms during daily activities; III - symptoms triggered by less intense activities than daily activities or by small efforts; IV - symptoms upon minimal exertion or at rest.

### Pulmonary function

Spirometry was used to evaluate the lung limits and capacities of the participants included in the study.This test was evaluated using a plethysmograph (Masterscreen Body, Mijnhardt/Jäger, Würzburg, Germany) by a previously trained researcher using conventional techniques and following the technical acceptability and reproducibility recommendations of the American Thoracic and European Respiratory Societies (ATS/ERS) [24]. At least three slow, forced, acceptable and reproducible maneuvers were performed, repeated 20 min after inhalation of Albuterol Sulfate (400 µg).

### Transthoracic echocardiogram

Transthoracic echocardiogram was performed by a cardiologist, using an ultrasound device with a 3 MHz transducer (Phillips, HD11 XE, Bothell, *Washington*, United States) according to the recommendations [25]. The left ventricular (LV) end-diastolic diameter, LV end-systolic diameter volume was obtained. Color tissue Doppler imaging was performed to determine early (E wave) diastolic mitral filling velocities. In addition, early diastolic mitral annular velocity was obtained (E’ wave) and used to calculate the mitral E/E’. The left ventricular ejection fraction (LVEF) was calculated using the Simpson method.

### Participants follow-up

To track all-cause mortality among participants included in the study, semiannual telephone calls were made to the patient and/or guardian/family member after the date of the patient’s initial laboratory evaluation. The causes of death were identified according to the death certificate and categorized individually according to the record. Additionally, medical records from the recruitment sites were used to collect information and the obituary system of the city of São Carlos, SP, Brazil < https://www.saocarlosagora.com.br/obituarios/>.

### Statistical analyses

Data are presented as mean and standard deviation or absolute values and percentages of occurrence when appropriate or median and minimum and maximum value. To establish a cutoff point, the Receiver Operating Characteristic (ROC) curve analysis was used to discriminate the DASI questionnaire score in determining the risk of mortality. The choice of the cutoff point followed the frequently used criterion where specificity is similar to sensitivity [26]. AUC values were classified as follows: AUC < 0.5, low predictive capacity, 0.7 ≤ AUC < 0.8, good predictive capacity and 0.8 ≤ AUC < 0.9, excellent predictive capacity [27].The Kolmogorov-Smirnov test was used to verify data normality.

For analysis between groups (> 23 step points and ≤ 23 points), the Student’s t test was used when normality assumptions were met or the Mann-Whitney test was used when the distribution was not normal. For categorical variables, the χ^2^ test was used to compare variable values between groups.

To determine the optimal cutoff point, we calculate the Youden Index (sensitivity + specificity − 1) for each possible cutoff value, selecting the one that produces the maximum index value. This approach ensures that a cutoff point is chosen that optimally balances the sensitivity and specificity of the test, providing the best possible diagnostic performance [26]. This index varies from 0 to 1, where J = 1 represents perfect accuracy, that is, complete separation between survivors and non-survivors, and J = 0 represents complete overlap, reflecting the inability of the diagnostic marker to discriminate between groups. For survival analysis, the Kaplan-Meier model was used to explore the impact of ≤ 23 points on mortality occurring during the 36-month follow-up.

The differences between the curves were evaluated using the Log-rank, Breslow and Tarone-Ware tests, and a value of *p* < 0.05 was adopted as significant. A binary logistic regression analysis was performed to examine the association between Duke Activity Status Index (DASI) scores and the occurrence of deaths. The dependent variable was binary categorical, representing survival (0) and death (1), while the primary independent variable was the DASI score. The model fit was assessed using the Hosmer and Lemeshow test and a value of > 0.05 was used as the criterion for not rejecting the null hypothesis, suggesting that the model fits the data well.Cox & Snell and Nagelkerke R² measures were calculated to assess the proportion of variability explained by the model. Cox proportional regression models [adjusted for ejection fraction (%), age (years), DASI questionnaire (score) and digitalis (1, yes; 0, no)] were performed, with associations expressed as hazard ratios (HR) and 95%CI.

All analyzes were performed using the GraphPad Prism version 8.0.1 for Windows (GraphPad Software, Boston, Massachusetts USA), <www.graphpad.com. The probability of type 1 error occurring was set at 5% for all tests (*p* < 0.05).

## Results

**Fig. 1:**
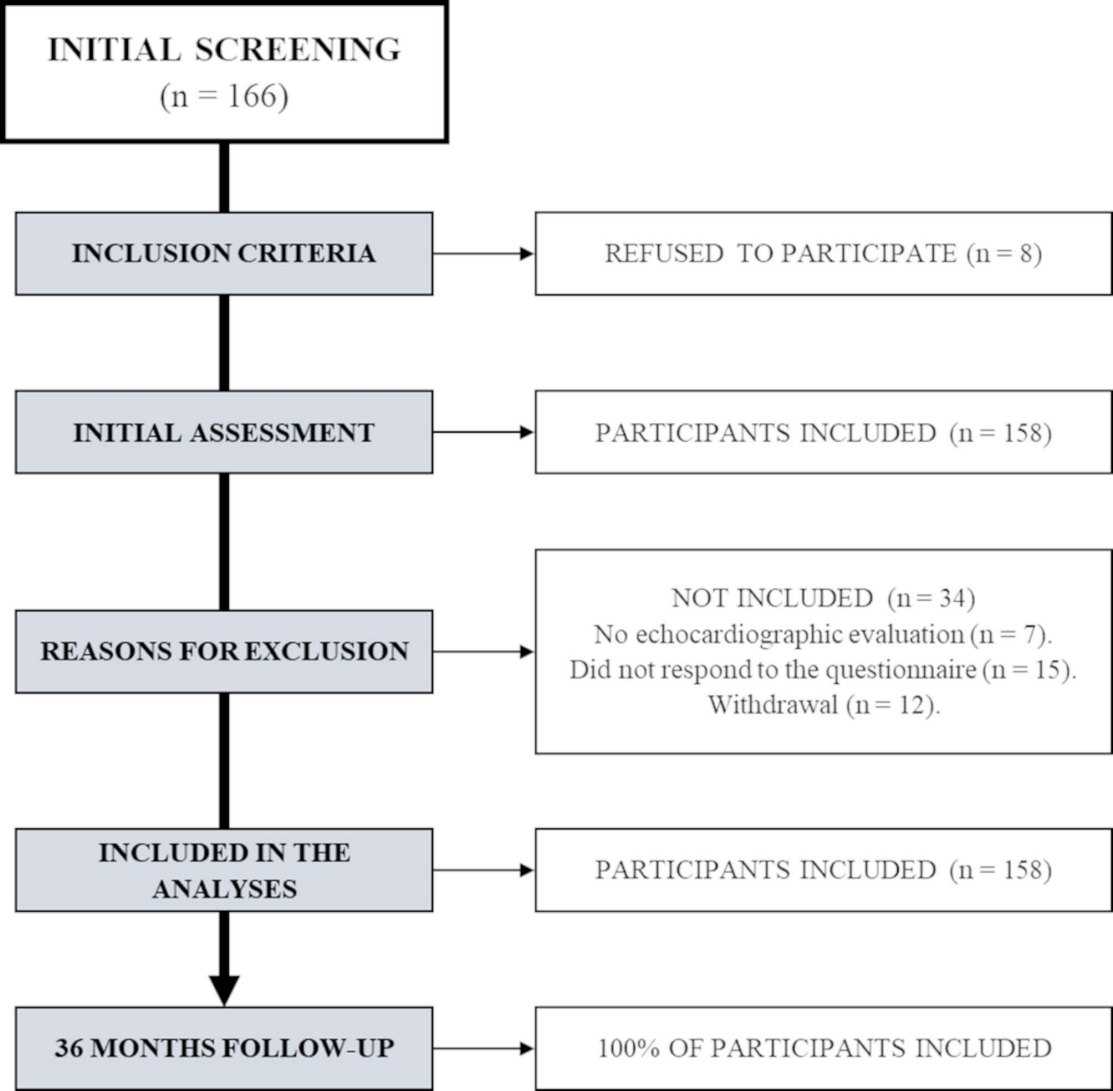
Flowchart study design including inclusion/exclusion criteria

Initially, one hundred and sixty-six patients were invited to participate in the investigation. However, forty-two patients were not included and the reasons can be seen in the flowchart illustrated in Fig. 1 - one hundred and twenty-four patients were included in the analysis. Table 1 contains information on the characterization of the sample, which was mainly composed of men (69%), with an average age of 62 years, overweight (39%) according to BMI (kg/m²), hypertensive (73%), ex-smokers (55%), dyslipidemia (47%) and type II diabetes (43%). The sample mainly included individuals with functional class I (36%) and II (36%) according to the NYHA scale. A large part of the sample (55%) had mild ejection fraction dysfunction according to the echocardiogram results, and the main medications used were beta blockers (83%), antihypertensive (73%) and anticoagulants (62%).The main cause of death was decompensation of heart failure (73%).All 124 participants included in the analyzes were adequately followed for 36 months.

**Table 1:** General characteristics of the study sample (*n* = 124)

Variables	CHF (*n* = 124)	≤ 23 points (*n* = 49)	˃23 points(*n* = 75)	*P* value
Age (years)	62 ± 11	62 ± 11	61 ± 11	0.783
Gender				
Male, n (%)	86 (69)	23 (47)	63 (84)	< 0.001*
Female, n (%)	38 (31)	26 (53)	12 (12)	
Total Body Mass (kg)	79.04 ± 17.17	82.24 ± 16.55	76.95 ± 17.34	0.094
Height(m)	1.66 ± 0.09	1.65 ± 0.09	1.66 ± 0.09	0.686
BMI (kg/m²)	28.61 ± 5.76	29.93 ± 5.60	27.75 ± 5.75	0.039*
Normal	31 (25)	7 (14)	24 (32)	0.271
Overweight	48 (39)	21 (43)	27(36)	
Obesity class I	29 (23)	13 (27)	16 (21)	
Obesity class II	12 (10)	6 (12)	6 (8)	
Obesity class III	3 (3)	2 (4)	2 (3)	
Body Fat Mass (kg)	27.16 ± 10.99	29.10 ± 11.02	26.37 ± 11.01	0.366
Body fat (%)	32.48 ± 8.87	34.94 ± 8.84	31.44 ± 8.78	0.162
Skeletal Muscle Mass (kg)	31.30 ± 9.19	28.61 ± 8.66	32.35 ± 9.27	0.145
Follow-up time (months)	32 (2–36)	36 (2–36)	36 (6–36)	< 0.001*
Death patients, n(%)	29 (23)	21 (43)	8 (11)	< 0.001*
Decompensation of Heart Failure	21 (73)	16 (75)	5 (61)	
Acute myocardial infarction	2 (7)	1 (5)	1 (13)	
Cancer	2 (7)	2 (10)	0 (0)	
COVID-19	2 (7)	1 (5)	1 (13)	
Complications of Diabetes Mellitus	1 (3)	1 (5)	0 (0)	
Kidney failure	1 (3)	0 (0)	1 (13)	
Risk factors, n (%)				
Asma	14 (11)	8 (16)	6 (8)	0.147
Atherosclerosis	3 (2)	1 (2)	2 (3)	0.829
Coronary artery disease	7 (6)	2 (4)	5 (7)	0.548
Hypertension	90 (73)	32 (65)	58 (77)	0.151
Depression	27 (22)	13 (27)	14 (19)	0.200
COPD	23 (19)	8 (16)	15 (20)	0.607
Obesity	44 (35)	21 (43)	24 (32)	0.219
Dyslipidemia	58 (47)	23 (47)	35 (47)	0.947
Deep vein thrombosis	7 (6)	4 (8)	3 (4)	0.321
Stress	27 (22)	8 (16)	19 (25)	0.242
Type 2 Diabetes	53 (43)	26 (53)	27 (36)	0.054
Alcoholism	7 (6)	4 (8)	3 (4)	0.432
Thyroid Disease	17 (14)	4 (8)	13 (17)	0.150
Obstructive Sleep Apnea Syndrome	10 (8)	5 (10)	5 (7)	0.472
Current Smokers	22 (18)	6 (12)	16 (21)	0.200
Ex-smokers	68 (55)	26 (53)	42 (56)	0.877
DASI questionnaire	29.75 ± 14.55	15.46 ± 4.74	39.07 ± 10.67	< 0.001*
DASI questionnaire (VO_2_)	22.28 ± 7.75	16.25 ± 2.50	26.22 ± 7.22	< 0.001*
Minnesota questionnaire	32.00 ± 22.00	44.00 ± 19.00	23.00 ± 19.00	< 0.001*
NYHA, n (%)				
I	44 (36)	3 (6)	41 (55)	< 0.001*
II	45 (36)	23 (47)	22 (29)	
III	29 (23)	20 (41)	9 (12)	
IV	6 (5)	3 (6)	3 (4)	
Pulmonary Function				
FEV1 (L)	2.39 ± 0.34	2.11 ± 0.53	2.54 ± 0.80	0.005*
FEV1 (%)	83.88 ± 20.50	79.13 ± 18.10	86.33 ± 21.34	0.091
FVC (L)	3.27 ± 0.96	2.82 ± 0.68	3.50 ± 1.00	< 0.001*
FVC (%)	91.15 ± 18.12	87.49 ± 18.87	93.03 ± 17.55	0.142
FEV1/FVC	0.74 ± 0.12	0.77 ± 0.05	0.71 ± 0.15	0.020*
Echocardiogram				
LV end-diastolic diameter (mm)	47.10 ± 11.36	51.15 ± 12.56	44.42 ± 9.70	0.003*
LV end-systolic diameter (mm)	58.31 ± 10.82	62.11 ± 12.01	56.16 ± 19.53	0.006*
Mitral E wave (cm/s)	71.86 ± 24.64	76.78 ± 24.43	68.58 ± 24.48	0.146
Mitral E’ wave (cm/s)	7.26 ± 2.67	7.22 ± 2.62	7.29 ± 2.74	0.927
E/e’ ratio	9.19 ± 6.55	10.89 ± 7.73	6.64 ± 3.01	0.083
LVEF, %	38.03 ± 9.69	34.41 ± 10.67	40.40 ± 8.24	0.001*
Medications, n (%)				
SABA	8 (7)	4 (8)	4 (5)	0.523
LABA	7 (6)	3 (6)	4 (5)	0.845
LAMA	2 (2)	1 (2)	1 (1)	0.756
Bronchodilator	16 (13)	9 (18)	7 (9)	0.138
Antihypertensive	90 (73)	35 (71)	55 (67)	0.986
Anticoagulant	77 (62)	26 (53)	51 (68)	0.130
Digoxin	29 (23)	20 (41)	9 (12)	0.026*
Beta blocker	103 (83)	43 (88)	60 (80)	0.117
Statins	55 (44)	17 (35)	38 (51)	0.102

Values are mean ± SD, absolute values (%)or median (minimum and maximum value). CHF: chronic heart failure; %: percentage; kg: kilos; m: meter; BMI: body mass index; COPD: Chronic Obstructive Pulmonary Disease; DASI: Duke Activity Status Index; VO2: oxygen uptake; NYHA: New York Heart Association; FEV_1_: forced expiratory volume in 1 s; L: liters; FVC: forced vital capacity; LV: left ventricular; millimeter; cm: centimeter; Mitral E/E’ ratio: early diastolic mitral filling velocity/ early diastolic mitral annular velocity; LVEF: left ventricular ejection fraction; SABA: short-acting β-agonist; LABA: long-acting β-agonist; LAMA: long-acting muscarinic antagonists. *p < 0.05 Statistical significance for Student’s t-test, Mann-Whitney test or χ^2^ test

**Fig. 2:**
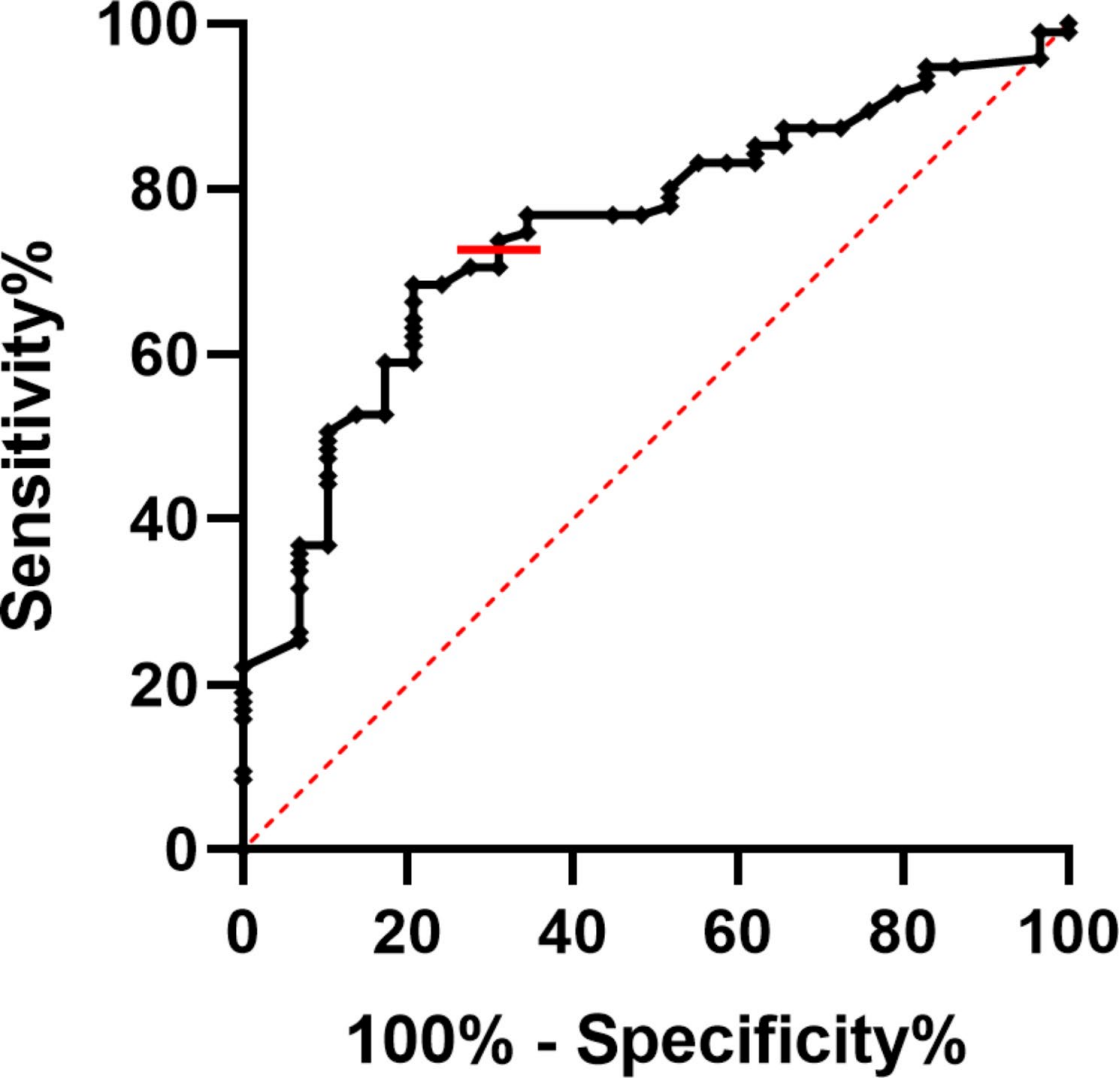
Receiver operating curves and AUC of DASI questionnaire and mortality over 36 months in CHF. AUC: area under curve; DASI: duke activity status index, CHF: chronic heart failure. The red line illustrates the best cutoff point on the graph

Based on the analysis of the ROC curve (Table 2; Fig. 2), the optimal cutoff values for achieving ideal sensitivity and specificity were determined to be ≤/> 23 points on the DASI questionnaire. This yielded a sensitivity of 70.53%, a specificity of 68.97% and a Youden index of 0.40.

**Table 2:** Cutoff value, sensitivity and specificity of DASI questionnaire scores for mortality in CHF patients

Cutoff	Sensitivity (%)	Specificity (%)	AUC [CI – 95%]	Youden Index	Positive Likelihood	Negative Likelihood
≤ 23	70.53	68.97	0.749 [0.656–0.843]	0.40	2.27	0.43

DASI: Duke Activity Status Index; ≤: less than or equal to; %: percentage; AUC: area under curve; CI: confidence interval

Comparative analysis between groups (Table 1) revealed that the group with the lowest score had a significant higher percentage of female patients (53%), overweight (43%), cases of death (43%) and patients using digitalis(41%). Not surprisingly, this same group presented lower functional capacity according to peakV̇O_2_ estimated by the DASI questionnaire, and the MLHFQ revealed a poorer quality of life. Lung function was significantly worse in the group with the worst performance on the questionnaire, as was LVEF. In this same group, a greater number of participants used the digitalis (41%) when compared to the group with better performance on the questionnaire.

The results of binary logistic regression (Table 3) indicate that DASI questionnaire scores are significantly associated with mortality (β = -0.075; *p* < 0.001). The odds ratio (HR) for DASI scores was 0.928, with a 95% confidence interval between 0.892 and 0.966, suggesting that an increase in DASI scores is associated with a reduction in the likelihood of death.

**Table 3:** Binary logistic regression model to investigate the association between DASI questionnaire scores and mortality

Model	β	Standard Error	Wald	*P* value	HR	95% CI toHR	
						Lower	Upper
DASI scores	-0.075	0.020	13.381	< 0.001*	0.928	0.892	0.966
Constant	0.746	0.515	2.097	0.148	2.108		

Dependent Variable: death (0; no; 1, yes)

Hosmer and Lemeshow test: 0.436

R^2^ Cox & Snell: 0.135

R^2^ Nagelkerke: 0.204

DASI: Duke Activity Status Index; β: beta; HR: hazard ratio; CI: confidence interval; *Significance value: *p* < 0.05

**Fig. 3:**
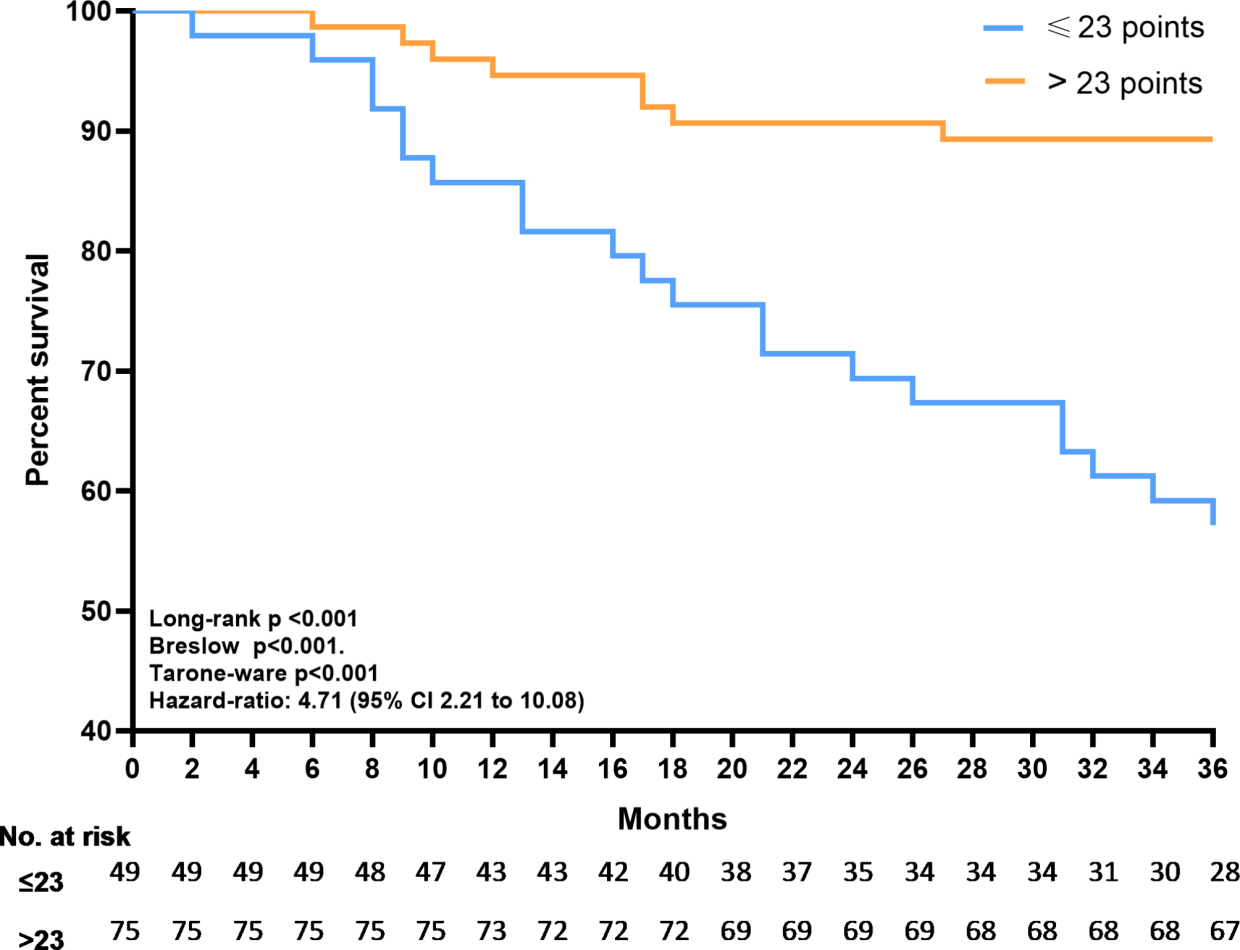
Kaplan-Meier survival analysis to assess mortality rates based on the DASI questionnaire using a cutoff point over a 36-month period. DASI: Duke Activity Status Index;> greater than; ≤less than or equal to; statistically significant difference for Log-rank, Breslow and Tarone-ware tests (*p* < 0.05)

Figure 3 illustrates the Kaplan-Meier curves over the 36-month follow-up period, revealing an increase in the risk of mortality over time in the group that presented with a DASI questionnaire score below the threshold by ROC analysis (Log-rank test, Breslow and Tarone-Ware *p* < 0.001; Hazard-Ratio: 4.71).When developing a predictive model for time-to-mortality data using Cox regression (Table 4), we observed that age (B = 0.044; 95%CI 1.010, 1.081), LVEF (%) (B = -0.042; 95% CI 0.925, 0.994), DASI score (B = -0.052; 95%CI 0.917, 0.983) and digoxin (-0.903, 95%CI 0.181, 0.909) were predictors of mortality risk.

**Table 4:** Risk factors for mortality in 124 patients with CHF

Covariates	B	SE	*P* value	Hazard Ratio	95%CI	
Ejection Fraction (%)	-0.042	0.018	0.022*	0.959	0.925	0.994
Age (years)	0.044	0.017	0.010*	1.045	1.010	1.081
DASI (score)	-0.052	0.018	0.004*	0.949	0.917	0.983
Digitals (1. yes; 0. no)	-0.903	0.412	0.029*	0.405	0.181	0.909

DASI: Duke Activity Status Index questionnaire; CHF: chronic heart failure; %: percentage; SE: standard error; CI: confidence interval; *significance statistical (*p* < 0.05)

## Discussion

The main findings of this investigation are: (1) a cutoff point of ≤/>23forthe DASI questionnaire score can predict mortality in individuals with CHF; and (2) In a multivariate model, age, LVEF, the DASI questionnaire score and use of digitalis are significant predictors of mortality. Interestingly, our sample was composed of patients with CHF and reduced (< 40%) or borderline (< 55%) LVEF. It is important to address the discriminatory capacity of the DASI questionnaire regarding mortality risk. Taking our results into account, sensitivity and specificity appear to be equivalent, however, the DASI questionnaire was found to be more sensitive than specific by a small percentage difference.

Undoubtedly, the assessment of exercise and functional capacity in the CHF population not only provides information about the ability of individuals to carry out the daily activities necessary to care for themselves [28], but is a key point in predicting unfavorable outcomes such as decompensation of pathology [29], necessity for hospitalization [29]and risk of mortality [30]. Main guidelines that guide CHF management reinforce the importance of evaluating this outcome using tools that have good discriminatory capacity, especially when correlated with CPET [31]. Although CPET is strongly recommended and should not be replaced, other approaches which are readily available and simple to administer, such as the DASI questionnaire [19], should be considered.

Above, we highlighted our results on the aspects of sensitivity and specificity, which is similar to the findings of Muatafaoglu et al. [12] in patients with pulmonary hypertension, where a DASI questionnaire cutoff point ≥ 26 points proved to have discriminatory capacity according to ROC analysis (AUC = 0.867), with a sensitivity of 0.74 and specificity of 0.88, capable of identifying patients with the best long-term prognosis according to functional capacity assessed by the six-minute walking test [12]. However, the study^9^did not assess the time frame for outcome. Additionally, other authors demonstrated that a cutoff point of < 33.8 for the DASI questionnaire was the best cutoff value to classify patients with pulmonary hypertension at different risks, with satisfactory sensitivity and specificity, and the authors justify that the difference in the cutoff value can be explained by different degrees of impairment of exercise capacity and that, despite the significant difference between cutoff points, the questionnaire is capable of stratifying risk in this population [32].

In another study, a DASI questionnaire cutoff point > 34 points was associated with reduced odds of myocardial injury within 30 days, mortality within 1 year, or new disability in individuals electing for non-cardiac surgery [15]. No information on sensitivity and specificity of the questionnaire was reported in that study^12^. This same cutoff point was used to investigate whether the DASI could predict complications within thirty days in patients undergoing colorectal surgery and the authors came to the conclusion that patients who reported lower DASI scores preoperatively were more likely to develop serious complications within 30 days after surgery [13].

The prognostic value of the DASI questionnaire has been previously explored in individuals with CHF [16]. The authors observed that there was an association between lower functional status measured by the DASI and increased risk of mortality over five years in individuals with CHF with preserved and reduced LVEF. However, there was no previously defined cutoff point and, in this way, the discriminative capacity of the instrument was not explored. However, the authors of that study^13^, when evaluating the score quartiles, observed that the lowest DASI score quartile (< 15.5) was able to predict 3.3 times higher risk of five-year mortality when compared to the highest DASI score quartile (> 42.70). Paradoxically, a significant number of participants died in all quartiles, especially in the quartiles with the lowest scores, and no statistical difference is reported in the number of events: 1st quartile (< 15.5) 193 deaths; 2nd quartile (15.5–26.1) 166 deaths; 3rd quartile (26.2–42.7) 120 deaths; 4th quartile (> 42.7) 77 deaths.

However, important and necessary attention should be paid when we view the value of the Youden Index in our results (0.40). This may compromise the ability to correctly identify positive cases (sensitivity) and the ability to correctly identify negative cases (specificity). Unfortunately, the studies discussed above do not include in their analyses the exploration of the Youden Index as a diagnostic performance tool and we were unable to project this result with previous results. In the real world, clinically this may generate certain impacts and we advise that clinical evaluation combined with other predictive instruments may be considered so that the main objective is to categorize the individual safely regarding their long-term mortality risk and contribute to better therapeutic optimization in order to avoid this outcome. The principle of multidisciplinary evaluation should be considered at all times [33].

The previous reasoning applies to the study developed by Senthong et al. [14]who also investigated the mortality risk in patients with peripheral arterial disease, however, only interquartile ranges were considered and no diagnostic performance analysis was used. Additionally, although the Kaplan-Meier analysis demonstrated a significant number of deaths over five years in the lowest DASI quartile (< 18.95 points), an important number of deaths was also observed in the other quartiles.

Cox regression found that age, DASI questionnaire score, LVEF and use of digitalis were independent factors for the risk of mortality in the current CHF cohort. According to previous findings, it is nothing new that advanced age increases the chance of death in individuals with CHF [34]. Paradoxically, other variables appeared as protective factors, that is, as the value increases, the chance of mortality risk decreases. A higher DASI questionnaire score reflects better exercise and functional capacity and, arguably, the higher the perceived exercise and functional capacity, the lower the chance of risk for unfavorable outcomes [12–15]Interestingly, the use of the digitalis also presented a protective factor and this prompted us to understand why, since the literature presents dichotomous results that sometimes attest to a reduced risk of mortality, at other times attest to an increased risk of this outcome in patients with CHF [35–37].

This brief overview of current literature encourages us to consider how sensitive and/or specific the DASI questionnaire can be compared to other numerous parameters that scientists propose to investigate and which, in short, reflect on the effectiveness and ability to adequately identify those at risk. Furthermore, these metrics are fundamental to the validity and clinical usefulness of the DASI questionnaire in predicting outcomes.

### Clinical impact

The DASI questionnaire is a simple, self-administered and easily accessible tool. Clinically, its applicability has been used to assess exercise and functional capacity as well as prognosis. This can assist health care professionals involved in the rehabilitation of CHF patients to obtain an overview of functionality, predict the risk of mortality and direct specific therapies so that this outcome can be avoided.

### Limitations

Our study has some limitations that should be considered. Firstly, participants who chose to leavethe study also chose to withdraw from the follow-up, and it was therefore not possible to censor them. Secondly, the initial project was designed to include only patients with a LVEF < 50%; therefore, patients with CHF and preserved ejection fraction were not included and findings presented here may not translate to the latter phenotype. Although DASI has a sensitivity and specificity of around 70% for mortality risk, the Youden Index has a limited value.

## Conclusion

A DASI questionnaire score of ≤ 23 presents good discriminatory capacity to predict mortality risk in patients with CHF with reduced or borderline LVEF. Age, LVEF, DASI questionnaire score and use of digitalis are risk factors that influence mortality.

## Data Availability

The datasets used and/or analyzed during the current study are available from the corresponding author on reasonable request.
